# RD-RAP: beyond rare disease patient registries, devising a comprehensive data and analytic framework

**DOI:** 10.1186/s13023-019-1139-9

**Published:** 2019-07-12

**Authors:** Matthew I. Bellgard, Tom Snelling, James M. McGree

**Affiliations:** 1 Office of eResearch, Queensland University of Technology, Brisbane, 4000 Australia; 2 Wesfarmers Centre of Vaccines & Infectious Diseases, Telethon Kids Institute, Perth, 6009 Australia; 3 School of Mathematics, Queensland University of Technology, Brisbane, 4000 Australia

**Keywords:** Rare disease, Patient registries, Base line data, Clinical decision making, Health outcomes, Analytics

## Abstract

Within the 21 APEC economies alone, there are an estimated 200 million individuals living with a rare disease. As such, health data on these individuals, and hence patient registries, are vital. However, registries can come in many different forms and operating models in different jurisdictions. They possess a varying degree of functionality and are used for a variety of purposes. For instance registries can facilitate service planning as well as underpin public health and clinical research by providing de-identified data to researchers. Furthermore, registries may be used to create and disseminate new knowledge to inform clinical best practice and care, to identify and enrol participants for clinical trials, and to enable seamless integration of patient data for diagnostic testing and cascade screening. Registries that add capability such as capturing patient reported outcomes enable patients, and their carers, to become active partners in their care, rapidly furthering research and ensuring up-to-date practice-based evidence. Typically, a patient registry centres around the notion of health data ‘capture’, usually for only one or a small subset of the functions outlined above, thereby creating fragmented datasets that, despite the best efforts and intentions, make it difficult to exchange the right data for the right purpose to the right stakeholder under appropriate governance arrangements. Trying to incorporate maximum functionality into a registry is an obvious strategy, but monolithic software solutions are not desirable. As an alternative, we propose that it is important to incorporate analytics as core to a patient registry, rather than just utilising registries as a ‘data capture’ solution. We contend that embracing an analytics-centric focus makes it reasonable to imagine a future where it will be possible to evaluate the individual outcomes of health interventions in real time. The purposeful and, importantly, the repurposable application of health data will allow stakeholders to extract, create and reuse knowledge to improve health outcomes, assist clinical decision making, and improve health service design and delivery. To realise this vision, we introduce and describe the concept of a Rare Disease Registry and Analytics Platform (RD-RAP); one that we hope will make a meaningful difference to the lives of those living with a rare disease.

## Background

Rare diseases are a global health challenge as they are typically lifespan diseases and genetic in nature. As they are rare, there are only small numbers of patients in any one jurisdiction and there is limited prevalence and incidence data available for any given rare disease. Surprisingly, although individually rare, there are an estimated 200 million people living with one of approximately 7000 rare diseases within the 21 APEC economies alone [1]. Recognising the rare disease global challenge as part of its Healthy Asia Pacific 2020 Vision,^1^ in November 2018, all 21 APEC economies ratified the first ever APEC Rare Disease Action Plan.^2^ One of the key pillars of the Action Plan is to *Manage pooling and usage of patient data securely and effectively*. Members agreed that better use of patient data is key to addressing the challenges presented by rare diseases.

As there is a scarcity of outcome data for individuals living with a rare disease, the ‘lived experience’ data from patients/families are typically a rich and important source of information. The veracity of the captured data is important. Current approaches to the collection of data about rare diseases are housed in multiple disparate data repositories be they electronic health records, spreadsheets, registries, longitudinal databases, paper-based notes, and so forth. An important focus is on the collection of clinical, physiological, quality of life, patient-reported outcome data to inform decision making. Patient registries are typically used as the data store in an attempt to assimilate patient data from various sources. However, it is important to note that a registry can come in many different forms depending on how and why it is used by which health stakeholder.

For instance, a registry from a clinical perspective may be used to track therapies, clinically significant endpoints, or clinical trials. From a patient advocacy perspective, patient-reported outcomes are important for collecting quality of life data to assist in assessing and supporting the impact to families and carers. From a government perspective, understanding health services planning, health economic assessment, determining subsidies and cost-effective products as well as reducing variability in healthcare are critical. For industry, it is the cost effectiveness of outcomes of treatments over time, and finally, from an academic perspective, natural history studies and the natural progression of disease are essential to drive discovery and innovation. There is no shortage of the variety and volume of rare disease health data contained within registries of varying veracity. Rare disease data and analytics roadmaps for rare diseases have been proposed previously, for instance, [2].

In its broadest definition, a *registry* can refer to both software programs that collect and store data or the patient records that are so created. As highlighted in the preceding sections, a rare disease patient registry is usually more than a data store, depending on its purpose, but as noted elsewhere, there is no consistent definition of a patient registry in current use [3–5]. It is almost assumed that vital health data and registry functionality can become fragmented and siloed, institutional/regional-based, thereby rendering the registry systems limited in their usefulness. Herein lies a paradox. On the one hand, the value of rare disease data can only be maximised if data is not stored in siloed repositories. It is essential to make it possible for key health stakeholders to meaningfully analyse and interpret all available data for a range of purposes. On the other hand, registries are important because rare disease data must be constantly assimilated so that it becomes possible to capture the evolving disease progression and management. In other words, when it comes to rare disease patient registries, it is important to leverage the useful aspects whilst minimising the unintended inaccessibility of data.

An obvious solution might be to attempt to build a ‘one-stop-registry’ solution that incorporates all required functionality. However, it has proved difficult to build a single solution as registries are dynamic ecosystems where functional requirements evolve over time [6]. An alternative strategy that we propose is to focus on analytics; design an analytic conceptual framework that is built around interoperable components for each form of required analysis for the rare disease patient data journey. We contend that there needs to be a shift from an individual data ‘collection’ to a comprehensive data ‘analytics’ mindset that can seamlessly support and drive healthcare delivery, service improvement and best practice adoption. Within this paradigm, it would be conceivable to cater concurrently for the needs of clinicians, patient representatives, government, data analysis specialists, eResearch and ICT specialists, nurses, administration and industry. An analytics-centric view of a registry is at the heart of this approach. With such a paradigm shift it would become possible to cater for functionality required to encourage and enable interactions, cross-talk and assess degree of overlap between each of the stakeholders as they evolve over time. In this manuscript, we introduce a high level coverage of the concept of a Rare Disease Registry and Analytics Platform (RD-RAP) for both providing baseline disease data and enabling innovative analytics that can be embedded into routine care and used to inform health services planning.

## RD-RAP: Rare Disease Registry and Analytics Platform

**Fig. 1:**
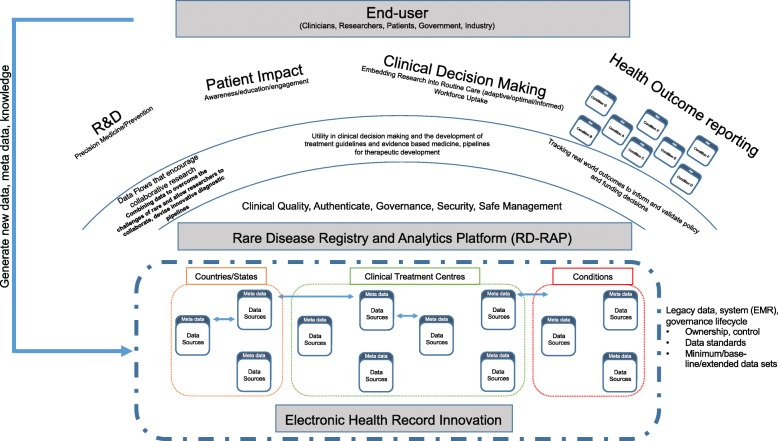
Conceptual Framework of the proposed Rare Disease Registry and Analytics Platform (RD-RAP)

Better use of patient data provides an opportunity to better support those living with a rare disease – enabling adaptive, optimal and informed decision making. For example, the right treatment, for the right patient and leveraging what has been learnt from past decisions to optimise future decisions. In rare diseases, there is inherent heterogeneity in the population such that individualised treatment and care is needed. However, of equal importance is building the evidence base for the rare disease population where it is critical to *learn* from individual experiences and aggregate these learnings across the rare disease population to find generality in disease progression, management and treatment. This duality of requirements, on more than one timescale, recognises the challenge to cater to individual needs as well as learn from collective experiences over time that will lead to better disease diagnosis and management, and personalised therapeutic interventions. It also highlights the challenges of designing patient registries fit for a single purpose. In order to devise sustainable and useful digital health solutions that address this duality it is important to develop an analytic conceptual framework, which we term the Rare Disease Registry and Analytics Platform (RD-RAP), shown in Fig. 1.

There are four core components to the RD-RAP conceptual framework. Firstly, analysis of rare disease data within RD-RAP is captured under four broad themes, namely: i) research and development, to support both precision medicine and health services planning; ii) patient impact, to capture patient reported outcomes; iii) clinical decision making, through the optimisation and adaptive models for routine care; and iv) health outcome reporting, to inform rare disease policy and health services planning. Areas covered would include personalised treatments, diagnosis, management of disease, disease prevalence, natural history studies and progression of disease. Providing capability for interactive engagement across each analytic theme is a key innovative component to RD-RAP as it proactively promotes collaboration, sharing of pre-competitive data and knowledge to drive discovery and innovation.

A second key component of RD-RAP is the governance structures that need to be developed to support a sustainable RD-RAP. The governance structures need to be developed in consultation with key stakeholders with an interest in RD-RAP such as international leaders from the clinical and patient community, eResearch and data analytics experts and they need to meet regularly. A RD-RAP Steering Committee (SC) would be established tasked with overseeing the rollout and development of RD-RAP responsible for the overall initiative, including which rare diseases or groups of rare diseases will be prioritised (e.g. drawing on European reference networks dedicated to specific group of diseases),^3^ development of appropriate privacy principles/practices for data collection, interoperability principles for both new and existing registries [7–9] storage, access and analytics arrangements for stakeholders, an operating model for sustainability and independence [for example, 10]. The SC will drive and leverage from international efforts (e.g. EU RDConnect^4^) to develop common datasets across conditions or, where common measures aren’t applicable, develop strategies to manage the data such as adopting the concept of registries within registries [6] which demonstrates a consent-driven approach to aggregate registries. The SC will participate in smaller work teams developed to undertake particular components of the project which include a review of issues of data ownership as well as related matters of legal frameworks and data security. A SC would be managed through an organisation such as the formalised APEC Rare Disease Network.^5^

A third key component to RD-RAP is that it recognises that electronic health records (EHR) or existing patient registries come in numerous forms and at varying stages of maturity globally. In addition, these systems might be jurisdiction-based, country-based, clinical centre-based or disease/conditions-based. For those living with a rare disease, in many instances, such systems may not exist in time for diagnosis, management or treatment. In addition, patients may not be able to utilise a quality EHR or get access to an existing international registry that is not available in a regional area. Furthermore a given rare disease may inhibit regular visits to a hospital where an EHR is available. For this reason, RD-RAP will be architected to enable automated and aggregated exchange of data between RD-RAP, electronic health records (EHR) and existing patient registries where feasible. This exchange will evolve over time and will necessarily capture data beyond what is captured within any given electronic capture system, driven by the priorities of government, industry, disease patient advocacy groups and clinicians requiring advanced analytics functionality across each of the four analytic themes. As outlined in Recommendation 9.3 of the APEC RD Action Plan,^6^ APEC member economies will facilitate cross-border data flows while respecting data privacy and applicable domestic laws and regulations. This will be achieved through four action steps, namely i) by leveraging partnerships with industry, clinicians, and patient organizations to design and implement an enabling environment for sharing patient data; ii) by ensuring full and informed consent from patients and families, interoperability of digital systems, and public availability of some data as permitted by local privacy and security contexts; iii) by adjusting as required by the Good Data Privacy Regulations (GDPR) and Cross-Border Privacy Regulations (CBPR); and iv) by working with academia to pool trial data related to small patient cohorts across jurisdictions.”

The fourth key component of RD-RAP is that this conceptual framework will provide a basis to work closely to support the implementation of suitable future-proofed digital health options for each of these stakeholders which is expected to evolve over time. Stakeholder consultations scoping RD-RAP technical and analytic requirements will ensure the RD-RAP meets the needs of key diverse stakeholders and this is fundamental to its success. This component will include working with governments, clinicians, researchers, patients and industry to identify current and future requirements regarding: i) data needs, including potential information sources, access, collection and collation; ii) data analytic needs, including analysis, design, diagnostics, therapeutics and economics; and technical e-Research specifications to ensure RD-RAP meet these needs. Once conducted, this input will enable the collection of essential baseline rare disease data and innovative analytics that will serve the needs of government, clinicians, researchers, patients and industry in assessing the health and economic benefits of proposed pharmacological treatments, clinical decision making, research and better patient management. These requirements will provide important feedback to enablecontinuous improvement of the health system to support those living with a rare disease.

### RD-RAP preliminary scoping activities

Design and development of the RD-RAP will employ best of breed eResearch technology adopting and developing open data standards, be open source and enable interoperability between developed digital health solutions. To realise the RD-RAP vision, RD-RAP will leverage exemplar national and global rare disease activities that are: i) developing open source digital health solutions to support N-of-1 adaptive clinical trials and patient reported outcomes; ii) analysis of clinical trial and observational study data and methodologies for designing and analysing adaptive trials; iii) diagnostics for detecting novel mutations and structural variants; iv) advocate therapeutics, clinical trials and regulatory harmonisation to map a pipeline into RD-RAP that will enable small to medium sized enterprises to work with rare-disease patient groups to evaluate their products; and v) promote patient engagement in all aspects of RD-RAP and research including patient registries, drug development, clinical trial design and recruitment, patient-reported outcome development, and real-world data collection and analysis.

On this last point, the activities for patient engagement are to identify, consult, and engage relevant patient associations and groups (national and multinational rare disease-specific groups as well rare disease alliances), to provide training and support to patients through patient groups to enhance interactions, contributions, and appropriate use of digital health platform and outputs, and to communicate with broader patient and public communities to promote understanding and support.

To work towards realising RD-RAP, currently an open source platform [6, 10–19] is being transformed to become a Trial-Ready Registry Framework (TRRF). TRRF is digital infrastructure to support adaptive clinical trials and ‘trial-ready’ natural history cohort studies. The open-source solution will enable seamless capture and linkage of clinician-entered and patient-reported data with health system administrative data, improving efficiencies for assessing and connecting eligible patients to trials, supporting the efficient systematic capture of data for trials, and for enabling real-time Bayesian analysis for novel trial designs. It is specifically intended to facilitate capture of clinical evidence to inform the licensure and funding of new therapeutic products, supporting: i) Trial-ready cohorts: A network of secure and robust clinical and patient-centred disease registries built on a shared platform will facilitate the accurate documentation of natural history across a range of diseases and disease subgroups. These data will be used to accelerate the evaluation of future treatments by allowing trialists to augment clinical trial data with data from historical controls; and ii) Pre-licensure trials: The FDA’s Center of Drug Evaluation and Research (CDER) now advocates the use of adaptive methods in place of traditional trial designs to expedite the clinical development of new products such as Bayesian adaptive platform trials like I-Spy2 (neoadjuvant therapies for breast cancer) and GBM-AGILE (brain cancer), facilitating seamless transition between trial phases and implementation of adaptive routines requires data to be captured, entered, and available for frequent interim analyses in near real time; paper-based platforms are inadequate.

Importantly, TRRF will include the ability to run N-of-1 trials [20–24]. N-of-1 trials are multi-cycle, double blinded clinical trials of treatment effect within an individual patient. As each individual acts as their own control, they can provide strong evidence that a particular treatment is effective for a given individual. Such trials are particularly suited when there is significant variability in treatment response within the patient cohort, and may be applicable in situations where large-scale parallel group trials are not possible as is the case of rare diseases. Further, individual N-of-1 trials can be aggregated to assess evidence of treatment effect at the population level (akin to that provided by parallel group trials).

Incorporating N-of-1 trials for developing the RD-RAP will facilitate evidence-based, personalised medical decision making to ensure each individual can determine what treatment works for them. Further, the framework will be flexible in that other trial designs such as parallel group, single case experimental designs, and cluster or step wedge designs can also be handled. The near real-time analytic capabilities of the RD-RAP will also extend naturally to adaptive and platform trials [25, 26]. Thus, despite focussing on N-of-1 clinical trials, the RD-RAP will be applicable to a wide range of clinical trial designs and will be extendible to emerging practices in this field.

### Overview of the systemic changes concerning the rare diseases ecosystem in an APEC economy

A number of APEC economies acknowledged, through the Rare Disease Economy Landscape Survey Australia that informed the APEC RD Action Plan, a disjointed and siloed approach to rare disease data collection. Often datasets are localised within an individual institution or linked to a clinician or number of clinicians working together for research purposes. The intent and design of the RD-RAP is to overcome the issues created by this approach and allow them to be utilised by cross sector stakeholders not just within one jurisdiction but across borders, thereby avoiding the notion of *health data imprisonment*. This will address the most fundamental issue of rare disease data collection, that is the collection of enough data to power appropriate analysis.

### RD-RAP implementation considerations

As identified by the APEC Rare Disease Network, data collection in rare disease is often siloed and based on individual clinics or hospitals. It lacks common data sets across registries and an ability to combine data to provide the power required for proper analysis. Existing data infrastructure brings with it considerable costs both financially and through the required time and effort to establish and maintain small, bespoke registries.

In order for RD-RAP to operate effectively work will need to be done to align data sets across registries. The system will also need to be easily adaptable to account for the many and varied rare diseases that it will serve. Finally, in order to drive the maximum benefit, regulatory changes will be required to allow cross border flow of data.

The open source approach to developing modules for use in the RD-RAP will aide in the development of common data sets. As clinicians and researchers find a readymade data collection tool available it is expected they will adopt the solution as a means of reducing the financial cost and timeliness of developing a bespoke solution. Further to this, the ability to access a much larger data set will act as further incentive for adoption of the RD-RAP tool. This open source approach is also expected to aide in the adaption of modules to account for different types of rare disease. Again, adaption of modules rather than the creation of bespoke solutions will reduce cost and time in establishing data collection projects.

Regulatory change is an issue that may require a longer lead time to achieve. The APEC Rare Disease Network in its recently public APEC Rare Disease Action Plan have identified this issue and have already commenced activity aimed at achieving regulatory alignment across APEC. The Network has presented the concept to the APEC Regulatory Harmonisation Committee and is continuing to work with this Committee on the concept.

### RD-RAP adoption considerations

The design of the RD-RAP concept has been undertaken with the issues of adaptability and usage in mind. The proposed approach does not increase the resource demands to capture data from already existing requirements. In fact, the strength of the RD-RAP is that it seeks to supplement data collection at the clinic level by aggregating data from existing sources rather than requiring data to be entered into yet another system. This design element will address the common constraints on resources that exist in data collection.

## Conclusions

In this Position Statement manuscript, we present the Rare Disease Registry and Analytic Platform (RD-RAP) conceptual framework. We do not underestimate the enormity of the task to design and implement RD-RAP but wish to put into context rare disease requirements in terms of registries and analytics for the benefit of a more general audience of clinicians, researchers and health systems and policy experts. As such, discussion of technical details is beyond the scope of this manuscript. We believe, RD-RAP positions the rare disease community to be a patient-led, locally supported and globally enabled innovative digital and analytics initiative. The outcome will be a framework for the development and implementation of RD-RAP serving the needs of the diverse stakeholders to support those living with a rare disease. RD-RAP aims to shift the rare disease registry community from a ‘data collection’ to a ‘data analytics’ paradigm that supports and drives healthcare delivery, service improvement and best practice adoption. A multidisciplinary and international team including clinicians, patient representatives, government, nursing, data analytics, eResearch and ICT will be assembled to design a digital framework for the establishment of an independent rare disease registry and analytic platform (RD-RAP). This platform will provide for essential baseline rare disease data and innovative analytics – including data analysis, design, diagnostics, therapeutics and health economics – that will serve the needs of government, clinicians, nurses, researchers, patients and industry in assessing the health and economic benefits of proposed pharmacological treatments, clinical decision making, research and better patient management. The framework will draw on the APEC Rare Disease Network to build collaborations with regional partners for its implementation and will foster a greater understanding of rare diseases and their impacts on health and assist in the allocation of healthcare expenditure.

## Data Availability

Publicly available.

## Footnotes

1
https://www.apec.org/Groups/Committee-on-Trade-and-Investment/Life-Sciences-Innovation-Forum

2
https://www.apec.org/rarediseases

3
https://ec.europa.eu/health/ern/networks_en

4
https://rd-connect.eu/

5
https://www.apec.org/rarediseases

6
https://www.apec.org/rarediseases
